# The Nitroplast and Its Relatives Support a Universal Model of Features Predicting Gene Retention in Endosymbiont and Organelle Genomes

**DOI:** 10.1093/gbe/evae132

**Published:** 2024-06-20

**Authors:** Iain G Johnston

**Affiliations:** Department of Mathematics, University of Bergen, Bergen, Norway; Computational Biology Unit, University of Bergen, Bergen, Norway

**Keywords:** endosymbionts, organelles, genome evolution, genome erosion

## Abstract

Endosymbiotic relationships have shaped eukaryotic life. As endosymbionts coevolve with their host, toward full integration as organelles, their genomes tend to shrink, with genes being completely lost or transferred to the host nucleus. Modern endosymbionts and organelles show diverse patterns of gene retention, and why some genes and not others are retained in these genomes is not fully understood. Recent bioinformatic study has explored hypothesized influences on these evolutionary processes, finding that hydrophobicity and amino acid chemistry predict patterns of gene retention, both in organelles across eukaryotes and in less mature endosymbiotic relationships. The exciting ongoing elucidation of endosymbiotic relationships affords an independent set of instances to test this theory. Here, we compare the properties of retained genes in the nitroplast, recently reported to be an integrated organelle, two related cyanobacterial endosymbionts that form “spheroid bodies” in their host cells, and a range of other endosymbionts, with free-living relatives of each. We find that in each case, the symbiont's genome encodes proteins with higher hydrophobicity and lower amino pK_a_ than their free-living relative, supporting the data-derived model predicting the retention propensity of genes across endosymbiont and organelle genomes.

**Significance**

As endosymbionts evolve and become more and more linked with their hosts, their genomes are often “eroded,” with many of their original genes being lost. Why some genes are lost and some retained—even as these endosymbionts become integrated organelles—is still debated. In this note, we use data from recently reported organelles and endosymbionts to support a theory describing how properties of genes make them more or less likely to be retained through this erosion process.

## Introduction

Eukaryotic life has numerous independent examples of endosymbiotic relationships. These include integrated organelles like the mitochondrion and plastid acquired billions of years ago (Smith and Keeling 2015), through acquisition of a cyanobacterium around 100 million years ago to form the chromatophore in *Paulinella* algae (Gabr et al. 2020), to more recent acquisitions of bacterial endosymbionts in insects (Husnik and Keeling 2019). Other examples include the nitrogen-fixing endosymbiont in *Azolla* water ferns (Peters and Meeks 1989; Ran et al. 2010), a cyanobacterial symbiont of diatoms (Flores et al. 2022), a denitrifying endosymbiont in a ciliate host (Graf et al. 2021), “spheroid body” compartments in diatoms (Nakayama et al. 2011), and a nitrogen-fixing symbiont accompanying a picoeukaryotic alga (Thompson et al. 2012), which has since been characterized as an integrated organelle dubbed the “nitroplast” (Coale et al. 2024). In each of these cases, the proto-endosymbiont originally possessed a full genome. As endosymbiotic relationships proceed and endosymbionts become more and more integrated organelles in the host cell, the endosymbiont genome tends to become reduced, with genes completely lost or transferred to the host nucleus (Moran et al. 2009; McCutcheon and Moran 2012; Maier et al. 2013; Giannakis et al. 2022). In some cases, this process has been complete, leaving mitochondrion-related organelles with no mitochondrial DNA (Hjort et al. 2010; Makiuchi and Nozaki 2014). In other cases, a subset of genes is retained in the organelle or endosymbiont.

The retained subset of genes in organelles and endosymbionts varies dramatically across eukaryotes, and the features favoring gene retention are not completely understood (McCutcheon and Moran 2012; Smith and Keeling 2015; García-Pascual et al. 2022; Butenko et al. 2024; Giannakis et al. 2023, 2024). Reductive evolution has some similarities and some differences between bioenergetic organelles and other endosymbionts (Maier et al. 2013). Hypotheses for why some genes are preferentially retained have often focused on mitochondria and plastids and have included roles for hydrophobicity (making it harder for nuclear-encoded genes to be imported to the organelle; von Heijne 1986; Björkholm et al. 2015), favoring local individual control of organelles [colocalization for redox regulation (CoRR); Allen 2015], the economics of maintaining and expressing genes from different compartments (Kelly 2021), and others (quantitatively compared in Giannakis et al. 2022).

**Fig. 1.**
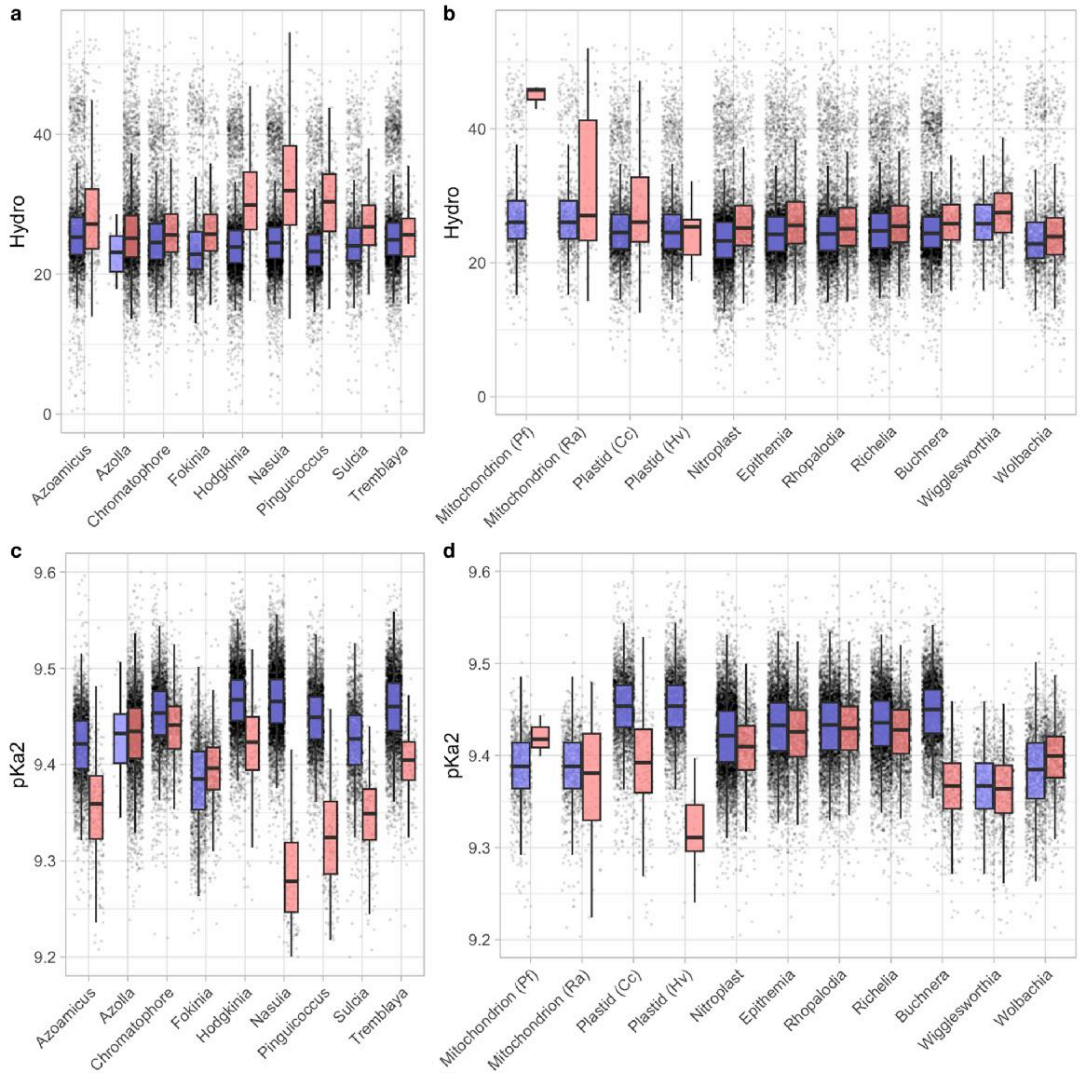
Differences between endosymbiont and free-living gene profiles consistently agree with model predictions. a and b) Hydrophobicity and c and d) amino pK_a_ distributions in genes retained in endosymbionts and organelles (red, right-hand bars) and free-living close relatives (blue, left-hand bars). a) was reported previously in Giannakis et al. (2022); c) is a new analysis of the source species from that publication; b–d) are newly analyzed here. Individual genes are shown as jittered points; boxplots give a summary distribution. Pf, *Plasmodium falciparum*; Ra, *Reclinomonas americana*; Cc, *Chondrus crispus*; Hv, *Hydnora visseri.*

Recent data-driven work has shown that models containing the same features (including hydrophobicity and acid dissociation constants) predict retention profiles in mitochondria and plastids across eukaryotes (Giannakis et al. 2022; Grub et al. 2022). Strikingly, when trained on mitochondria, this model predicts plastid retention patterns (and vice versa), suggesting that similar principles may shape gene retention in the two cases. Specifically, genes encoding products with high hydrophobicity and low amino pK_a_ were more likely to be retained, along with a role for the centrality of a protein subunit in its complex (related to CoRR). Hydrophobicity and pK_a_ values were also shown to differ systematically between other endosymbionts and their free-living relatives, in a set of relationships in insects, algae, and protists (Husnik and Keeling 2019; Fig. 1a).

The ongoing elucidation of examples along the spectrum from endosymbiont to mature organelle, including the nitroplast (Coale et al. 2024) and its cyanobacterial relatives (Nakayama and Inagaki 2017), allows an independent test of this “universal” model. In this note, we ask whether these other relationships, reflecting a spectrum of maturity of endosymbiosis, support this picture.

## Results

Here, we analyzed a collection of pairs of symbionts and free-living partners, including the nitroplast, spheroid body endosymbionts, and several other symbionts not explored in Giannakis et al. (2022). All organelles and symbionts newly considered showed substantial increased hydrophobicity compared with their free-living relatives (Fig. 1b). The spheroid bodies and *Richelia* showed a hydrophobicity increase on a similar scale to that seen in the *Paulinella* chromatophore (Fig. 1a). The increase was slightly greater in the nitroplast, on a similar scale to the nitrogen-fixing *Nostoc azollae* symbiont in the *Azolla* water fern (Fig. 1a).

Amino pK_a_ values were found to predict gene retention patterns in mitochondria and chloroplasts, but were not explicitly examined previously in other endosymbionts in Giannakis et al. (2022). Figure 1c shows the trends across the relationships explored in that study. With two exceptions (*Azolla* and *Fokinia*), amino pK_a_ values are lower (sometimes dramatically so) in endosymbionts than in free-living relatives, matching the behavior expected from the universal model. Plastids also show this behavior; the *Plasmodium* mitochondrion we consider instead has a higher average amino pK_a_. This is not inconsistent with the universal model picture: the very high difference in hydrophobicity in the *Plasmodium* mitochondria overcomes the pK_a_ term in the predictive model, so that the three genes are predicted to have a high retention index. In the set of newly considered relationships in this study (nitroplasts, spheroid bodies, and others), each endosymbiont (except *Wolbachia*, in the same family as *Fokinia*) also showed lower amino pK_a_ values than its free-living relative (Fig. 1d), again on a similar scale to the chromatophore, with this effect stronger for the nitroplast than for the spheroid bodies.

The gene-by-gene correlation across our data set of hydrophobicity and amino pK_a_ value is weak (*r*^2^ = 0.022), suggesting that Fig. 1a–d is not just reporting the same effect twice over; the behavior in hydrophobicity is largely independent on the behavior in pK_a_. This reflects the fact that in the original model selection process for organelle gene retention, the two features were selected together, suggesting that they provide independent information about gene retention propensity.

Significance testing for the individual comparisons in Fig. 1 is not directly meaningful, as the full sets of genes from each organism are being reported—there is no sampling noise to account for, so statements about mean differences are not subject to meaningful uncertainty. The more interesting hypothesis test relates to the observation of partnership comparisons, against the null hypothesis that hydrophobicity and pK_a_ do not differ between symbionts and relatives. If our symbiont–relative pairs are treated as independent, the probability of at least 13/14 new observations (7 partnerships, for hydrophobicity and pK_a_, with *Wolbachia* pK_a_ disagreeing with prediction) agreeing with the theory under the null hypothesis is *P*(*B* ≥ 13) for B ∼ Bin(14, 0.5), giving *P* = 9.2 × 10^−4^. If the two spheroid body partnerships are regarded as reflecting the same case, the probability becomes *P*(*B* ≥ 11) for B ∼ Bin(12, 0.5), giving *P* = 0.0032. The pairs are of course not truly independent, all being related to some extent, but the distance between most pairs is considerable.

Previous work has focused on ribosomal proteins in organelle and endosymbiont evolution (Maier et al. 2013). In supplementary fig. S1, Supplementary Material online, we show some aspects of the data set when proteins annotated as ribosomal and non-ribosomal are separated. In general, ribosomal proteins are less hydrophobic and have marginally lower amino pK_a_ values than other proteins (across symbionts and partners), reflecting their well-spread, cytosolic physical environment (supplementary fig. S1a and b, Supplementary Material online). In the case of the *Reclinomonas americana* mtDNA genome (supplementary fig. S1c, Supplementary Material online), there is a rather pronounced separation of proteins by hydrophobicity and pK_a_: one set of relatively hydrophilic, low pK_a_ proteins (dominated by ribosomal proteins) and one set of hydrophobic, high pK_a_ proteins (dominated by electron transport chain subunits); in other cases, there is a smoother spectrum of statistics (supplementary fig. S1b, Supplementary Material online). In most cases, symbiont–partner differences are conserved across ribosomal and non-ribosomal proteins.

## Discussion

From the study of mitochondria alone, a model involving hydrophobicity and amino acid biochemistry was found to predict gene retention patterns (Johnston and Williams 2016; Giannakis et al. 2022). The same model with the same parameters (positive effect for hydrophobicity, negative effect for amino pK_a_) also predicts plastid gene retention (Giannakis et al. 2022; Grub et al. 2022). We have found here that the same influences separate genes retained in endosymbionts across a range of maturities, from recent insect acquisitions to the more integrated and established chromatophore and nitroplast.

Why these features? Hydrophobicity was originally argued to challenge protein import to the organelle from the remote encoding of the nucleus (von Heijne 1986) and has since been suggested to influence mistargeting of protein products (Björkholm et al. 2015). In many of the relationships we consider, it is far from clear whether symbiont genes have been transferred to the nucleus, so whether hydrophobicity acts as a barrier to transfer is less well-posed. However, it can likely still act as a barrier to *loss*. All our cases do seem to involve reduction of the symbiont genome, likely due in part to redundancy, where host-encoded proteins can be used by the symbiont. For this to be the case, host-encoded proteins still require import to the endosymbiont, so the argument that hard-to-import machinery is more likely to be retained can still be used.

We previously and very speculatively suggested that links to pK_a_ could relate to the necessity of assembling proteins in a cellular compartment where pH may be different (Giannakis et al. 2022). pK_a_ reports how easily protons are lost from amino acids under different pH conditions and hence necessarily influences the dynamics of peptide formation in translation (Watts and Forster 2010). This influence leads to differences in peptide formation dynamics in different pH environments (Johansson et al. 2011). The differences in compartmental properties—including pH—as endosymbiotic relationships evolve could conceivably therefore mean that the inside-compartment ease of assembling proteins is greater for those with particular pK_a_ profiles. Once assembled, the pK_a_ profile of a protein dictates the ease of protonation in a basic (or acidic) environment, and if maintaining a certain protonation state is important for functionality, the compartmental pH may act to favor high (or low) pK_a_ values accordingly. However, further and more detailed investigation is needed to explore this hypothesis.

Of course, the consideration of two features alone cannot describe all the possible mechanisms and influences shaping endosymbiont genomes across relationships. The performance of models considering these features for mitochondrial and plastid gene retention is reasonable (Spearman's *ρ* around 0.5–0.6 for mtDNA and ptDNA genes outside the training sets; Giannakis et al. 2022), but the effect sizes are smaller in these less mature endosymbiotic cases, and the predictive power of such models will be more limited. There also appear to be systematic differences between the loss dynamics in bioenergetic organelles and other endosymbionts (Maier et al. 2013), as predicted by CoRR (Allen 2015). This note intends only to highlight that these exciting emerging cases provide further independent support for these features having some possible (not complete) influence over endosymbiont genome evolution, not that the question is resolved!

## Materials and Methods

Following the pipeline from Giannakis et al. (2022), we obtained coding sequence records for the collection of genomes in endosymbionts, organelles, and free-living relatives in Table 1. This set was originally chosen from a comprehensive review (Husnik and Keeling 2019); we included *Wolbachia* as a famous, though not obligate, endosymbiont example. Close free-living relatives were identified from phylogenetic analysis in the references cited therein and confirmed with NCBI Common Taxonomy Tree (Federhen 2012). For the Rickettsiales examples, most close relatives were also endosymbionts (often parasites), so we took statistics from a sister clade *Ca.* Pelagibacter ubique, the ubiquitous marine bacterium (Rappé et al. 2002). We also included mitochondria and chloroplasts from different species for comparison, compared with modern-day Rickettsia and cyanobacterial examples (Keeling 2010; Roger et al. 2017). We computed statistics for the protein corresponding to each gene in each record, specifically taking the mean hydrophobicity and mean carboxyl and amino pK_a_ values across amino acid residues in each sequence, using lookup tables from https://www.sigmaaldrich.com/NO/en/technical-documents/technical-article/protein-biology/protein-structural-analysis/amino-acid-reference-chart. Ribosomal identity was taken directly from the gene annotation in each case. Analysis was performed in Biopython (Cock et al. 2009) and R (R Core Team & Team, 2022) with libraries *ggplot2* (Wickham 2016) and *ggpubr* (Kassambara 2020) for visualization. Code for the analysis and visualization is freely available at https://github.com/StochasticBiology/endosymbiont-gene-loss.

**Table 1:** Pairs of endosymbionts and free-living relative, and organelles and non-organelle relatives, used for comparison in this study, with NCBI accessions and references supporting the choice of relative

Endosymbiont/organelle	Free-living/non-organelle relative	Notes and references
Mitochondrion (*R. americana* and *Plasmodium falciparum*; NC_001823.1 and NC_037526.1)	*Rickettsia typhi* (CP003398.1)	Bacterial-derived organelle found across almost all eukaryotes (Smith and Keeling 2015; Roger et al. 2017)
Plastid (*Chondrus crispus* and *Hydnora visseri*; NC_020795.1 and NC_029358.1)	*Synechococcus* PCC 7002 (CP000951)	Bacterial-derived organelle found across photosynthetic (and other) eukaryotes (Keeling 2010; Smith and Keeling 2015)
*Paulinella* chromatophore (CP000815.1)	*Synechococcus* PCC 7002 (CP000951)	Cyanobacterium-derived organelle in an alga (Lhee et al. 2019)
Nitroplast (UCYN-A, *Ca. Atelocyanobacterium thalassa*; CP001842.1)	*Crocosphaera watsonii* (GCF_000235665.1)	Nitrogen-fixing organelle in algae (Thompson et al. 2012; Coale et al. 2024)
*Epithemia turgida* spheroid body (AP012549)	*Rippkaea orientalis* (GCF_000021805.1)	Cyanobacterium-derived compartment in diatom (Nakayama and Inagaki 2017); closely related to *Rhopalodia gibberula* spheroid body and related to nitroplast (Qiu et al. 2021)
*R. gibberula* spheroid body (AP018341.1)	*Cyanothece* sp. PCC 8801 (CP001287.1)	Cyanobacterium-derived compartment in diatom (Nakayama and Inagaki 2017); closely related to *E. turgida* spheroid body and related to nitroplast (Qiu et al. 2021). *Rippkaea* is a free-living relative; comparison with another free-living relative *Cyanothece* is included to link with Giannakis et al. (2022)
*Ca.* Azoamicus ciliaticola (NZ_LR794158.1)	*Legionella clemsonensis* (NZ_CP016397)	Denitrifying endosymbiont in an anaerobic ciliate (Graf et al. 2021); most relatives, including *Legionella*, are largely intracellular
*N. azollae* (CP002059.1)	*Raphidiopsis brookii* (ACYB01000001.1)	Nitrogen-fixing cyanobacterium in a water fern (Ran et al. 2010)
*Richelia intracellularis* (GCA_000350105.1)	*Richelia sinica* (GCF_019056575.1)	Cyanobacterial symbiont in diatom (Flores et al. 2022)
*Nasuia deltocephalinicola* (CP013211.1)	*Herbaspirillum seropedicae* (CP002039.1)	Bacterial endosymbiont of insects (Bennett and Moran 2013)
*Ca.* Sulcia muelleri (CP001981.1)	*Porphyromonas gingivalis* (AE015924.1)	Bacterial endosymbiont of insects (McCutcheon and Moran 2007); “free-living” relative does invade cells but can survive independently in oral cavity
*Ca.* Tremblaya phenacola (CP003982.1)	*Sodalis praecaptivus* (CP006569.1)	Bacterial endosymbiont of insects (Enomoto et al. 2017)
*Ca.* Hodgkinia cicadicola (CP008699)	*Rhizobium etli* (CP007641.1)	Alpha-proteobacterial symbiont of cicadas (McCutcheon et al. 2009)
*Ca.* Pinguicoccus supinus (CP039370.1)	*Coraliomargarita akajimensis* (CP001998.1)	Bacterial endosymbiont in ciliate (Serra et al. 2020); partner is not the closest sequence found, but is the closest annotated sequence in putative phylogeny
*Ca.* Fokinia solitaria (CP025989.1)	*Ca.* Pelagibacter ubique (CP000084.1)	Rickettsiales endosymbiont (*Ca.* Midichloriaceae family) in ciliate (Floriano et al. 2018); like *Wolbachia*, all closest relatives are intracellular Rickettsiales—relative taken from a sister group
*Wigglesworthia glossinidia* (GCF_000247565.1)	*Pantoea agglomerans* (GCF_019048385.1)	Gammaproteobacterial endosymbiont of tsetse fly (Akman et al. 2002)
*Buchnera aphidicola* (GCF_003099975.1)	*P. agglomerans* (GCF_019048385.1)	Gammaproteobacterial endosymbiont of aphids (van Ham et al. 2003)
*Wolbachia pipientis* (GCF_014107475.1)	*Ca.* Pelagibacter ubique (CP000084.1)	Rickettsiales endosymbiont can exist as insect endosymbiont or independently (Werren et al. 2008); like *Fokinia*, all closest relatives are intracellular Rickettsiales—relative taken from a sister group

The species chosen for mitochondria and plastids correspond to very high (*R. americana*, *C. crispus*) and very low (*P. falciparum*, *H. visseri*) organelle DNA gene counts.

## Supplementary Material

evae132_Supplementary_Data

## Data Availability

NCBI accession numbers for all records used are given in Table 1. Code to reproduce the analysis and visualization is freely available at https://github.com/StochasticBiology/endosymbiont-gene-loss.
