# Supplementary material for: The Nitroplast and Its Relatives Support a Universal Model of Features Predicting Gene Retention in Endosymbiont and Organelle Genomes
Source: Genome Biol Evol. 2024 Jun 20;16(7):evae132. doi: 10.1093/gbe/evae132 (PMC11221429; doi:10.1093/gbe/evae132)
Supplement: evae132_Supplementary_Data [file evae132_supplementary_data.pdf]

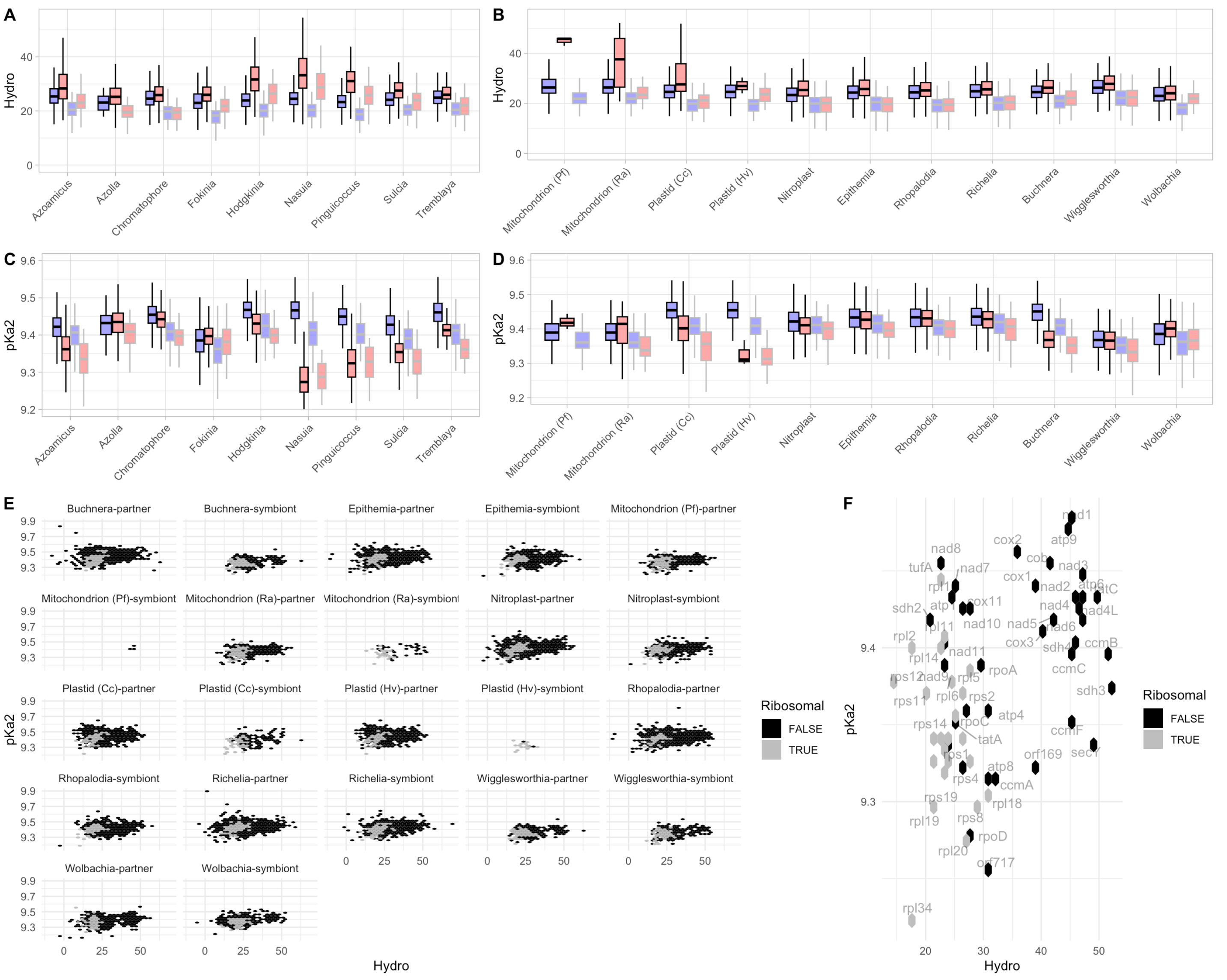

Supplementary Figure 1. **Comparing ribosomal and non-ribosomal annotated proteins.** (A-B) Hydrophobicity and (C-D) amino pK_a distributions in genes retained in endosymbionts and organelles and free-living close relatives, as in Fig. 1. For each pair, blue fills give free-living species; red fills give endosymbionts/organelles; black outline gives non-ribosomal proteins and gray outline gives ribosomal proteins. *Plasmodium* (Pf) mtDNA encodes no ribosomal proteins. (E) Joint hydrophobicity and amino pK_a distributions in each species. The plot label gives the system corresponding to the labels in A-D; "partner" is free-living partner, "symbiont" is symbiont/organelle. (F) Detail of joint hydrophobicity and amino pK_a distribution in *Reclinomonas americana* mtDNA genome, with individual genes labelled.
